# Computerized Dual-Task Testing of Gait and Visuospatial Cognitive Functions; Test-Retest Reliability and Validity

**DOI:** 10.3389/fnhum.2017.00105

**Published:** 2017-03-17

**Authors:** Tony J. Szturm, Vedant S. Sakhalkar, Anuprita Kanitkar, Mayur Nankar

**Affiliations:** ^1^Mobility and Cognition Lab, College of Rehabilitation Sciences, University of ManitobaWinnipeg, MB, Canada; ^2^Department of Physical Therapy, University of ManitobaWinnipeg, MB, Canada; ^3^School of Medical Rehabilitation, University of ManitobaWinnipeg, MB, Canada; ^4^Applied Health Sciences, University of ManitobaWinnipeg, MB, Canada

**Keywords:** treadmill walking, spatiotemporal gait variables, cognitive performance, Dual-task performance, intra-class correlation coefficient

## Abstract

The common occurrence of age decline in mobility and cognition does cause a decrease in the level of physical activity and an increased falls risk. Consequently, dual -task (DT) assessment that simultaneously addresses both mobility skills and cognitive functions are important because, continued difficulties and fall injuries will have a sizable impact in this population. The first objective of the present study was to assess test-retest reliability of a computerized DT treadmill walking protocol and concurrent outcome measures of gait and visuospatial executive function in a group of healthy older adults. Secondly, discriminative validity was evaluated by examining the effect of DT conditions (single task vs. dual-task) on; (a) spatiotemporal gait measures (average and coefficient of variation) and (b) visuomotor and visuospatial executive performance measures. Twenty-five community-dwelling individuals median age 65 (range 61–67) were recruited from a Fitness Facility. Participants performed a computerized visuomotor tracking task and a visuospatial executive game task in standing and while treadmill walking. Testing was conducted on two occasions, 1 week apart. Moderate to high test-retest reliability (ICC values of 0.65–0.88) were observed for spatiotemporal gait variables. No significant differences between the group means were observed between test periods in any gait variable. Moderate test-retest reliability (ICC values of 0.6–0.65) was observed for measures of visuomotor and visuospatial executive performance during treadmill walking. Significant DT effects were observed for both spatiotemporal gait variables and visuospatial executive performance measures. This study demonstrates the reliability and reproducibility of the computer-based assessment tool for dual task treadmill walking. The high to moderate ICC values and the lack of systematic errors in the measures indicate that this tool has the ability to repeatedly record reliable data from community-dwelling older adults. The present computerized dual-task protocols broaden the types of standardized visuomotor and visuospatial executive activities for use with DT treadmill walking that has previously been reported.

## Introduction

The frequent occurrence of mobility limitations and falls with age can arise due to singular events (e.g., stroke, peripheral vestibular dysfunction) or can have an insidious onset, with the problem source found in multiple predisposing factors, such as, the gradual decline of musculoskeletal and/or neural fitness (sensory, cognitive, motor; Santos-Eggimann et al., 2008). Walking problems and falls, in particular outdoors, become evident when compensatory strategies have failed, or where certain tasks and environmental conditions cannot be avoided (Shumway-Cook et al., 2007; Santos-Eggimann et al., 2008).

For older adults, community ambulation is strongly associated with the preservation of skills for independent living, community participation, and healthy aging (Simonsick et al., 2005). Safe, independent community walking outdoors requires both mobility skills and cognitive flexibility to address threats to balance while attending to a range of environmental demands and concurrent executive tasks. Mobility limitations and executive impairments common to aging often coexist and are prognostic of adverse health events, including falls (Santos-Eggimann et al., 2008; van Iersel et al., 2008; Herman et al., 2010; Ijmker and Lamoth, 2012). Consequently, dual- task screening and training programs that simultaneously address both mobility and cognition are important to consider in the promotion of healthy aging and in rehabilitation (Pichierri et al., 2011; Diamond, 2015; Gregory et al., 2016).

The application of digital media and computer technologies provides a number of promising approaches for dual task (DT) assessments and training. For example, a Virtual reality (VR) environments viewed during treadmill walking have been used to provide a more ecological and task-oriented approach to mobility training. Preliminary results suggest that mixed, augmented VR environments that incorporate both treadmill walking and executive tasks have the potential as a rehabilitation tool. (Mirelman et al., 2013; Park et al., 2015) The application of computer games has also received considerable interest from researchers and clinicians as a method to challenge and train many different aspects of executive functions (Anguera et al., 2013; Wolinsky et al., 2013; Rebok et al., 2014; Strenziok et al., 2014). These emerging rehabilitation technologies have the potential to improve clinical outcomes by making therapies and exercise more engaging more motivating and more effective. For this purpose, an engaging, Game-Based Rehabilitation Platform (GRP) for dual-task training with embedded assessment was developed (Szturm et al., 2013a,b). The platform provides an integrated approach to decline in balance, mobility, visuomotor and gaze control, and visuospatial executive function. The GRP consists of a standard treadmill instrumented with a pressure mapping system to record center of foot pressure, and an interactive computer game subsystem (Betker et al., 2008; Szturm et al., 2015a). The treadmill is equipped with a standard LED monitor, and thus, a broad range of visuomotor and visual-spatial executive game activities can easily be managed concurrently while treadmill walking. The GRP includes a monitoring application which uses advanced data logging and analysis method to record the client's actions and choices while playing designed rehabilitation assessment games. The game activities involve visual attention, visual search and tracking of moving visual targets, and the ability to select and interact with relevant targets and ignore/avoid distracter objects. Visuospatial processing is an important aspect of cognition to explore as a factor involved in age decline in mobility and increased fall risk (Santos-Eggimann et al., 2008; van Iersel et al., 2008; Nagamatsu et al., 2009; Murray et al., 2010). With this method both gait and visuospatial executive performance can be quantified during steady state walking at a constant velocity and over durations of 1–2 min or more, as tolerated (i.e., 40 to hundreds of consecutive steps).

Many over ground walking studies examine how information processing load affects gait rhythm or stability, (i.e., spatiotemporal gait variables (Al-Yahya et al., 2011). However, gait speed is a confounding variable, as spatiotemporal gait variables are sensitive to changes in gait speed (Kang and Dingwell, 2008; Stoquart et al., 2008; Szturm et al., 2013a; Keene et al., 2016). Most of over ground walking studies use an instrumented walkway, which records only 4–6 consecutive steps. This method may reliably measure gait speed, but is not sufficient for measures of gait variability or periodicity, particularly during dual-task walking (Bruijn et al., 2009; Hollman et al., 2010; Galna et al., 2013).

The purpose of this study is to establish the psychometric properties of the test protocols and the dual-task outcome measures of the GRP during treadmill walking. This is an important initial step before it can be routinely used clinically or in community centers for screening, fall risk assessment, and rehabilitation, as well as for preventative measures. Although, the test-retest reliability of mean values of spatiotemporal gait parameters has been assessed for reliability while walking alone, little is known about the test-retest reliability of gait variability while performing concurrent executive tasks (Brach et al., 2008; Paterson et al., 2008; Beauchet et al., 2011; Faude et al., 2012).

The first objective was to establish test-retest reliability of outcome measures that represent gait performance, visuomotor (VM) performance, and visuospatial executive function when tested during dual-task conditions. The second objective was to examine the discriminative validity of the computerized outcome measures. Specifically to examine the influence that information processing load has on gait function, and vice versa to examine the influence that physical demands of walking have on visuospatial cognition. A better understanding of the interactions between physical demands of walking and visuospatial cognition will be important for identifying high-risk scenarios that people might encounter outside the lab, and for designing effective, personalized exercise programs suitable for community applications.

## Methods

### Participants

Twenty-five adults participated, 19 male and 6 female, the median age of 65 years (range 60–67) who attended a Medical Fitness Facility. The participants were able to walk outside without any walking aids and had no self-reported history of falling. Exclusion criteria included histories of neurological or musculoskeletal disorders (e.g., stroke, hip/knee joint surgery). All participants provided written consent. The study was approved by the University of Manitoba research ethics committee. Prior to testing, each participant completed a 6-min walk test on a 300-m track, and the average walking speed was determined over a 25-m distance.

### Tests and instrumentation

**Figure 1:**
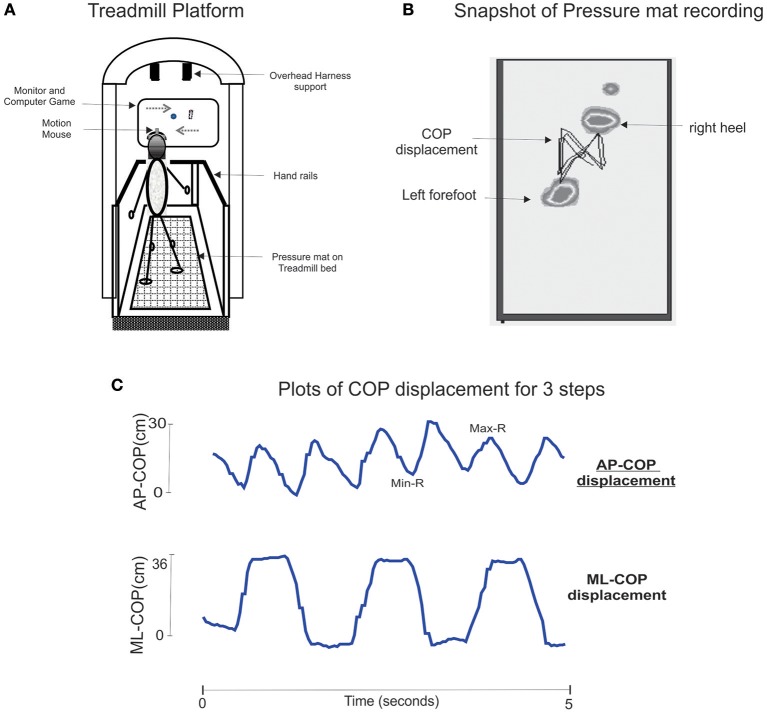
**Panel (A)** is an illustration of the treadmill platform and experimental set-up. Participant is walking on the instrumented treadmill while viewing a computer monitor. Head rotation (via motion mouse) is used to interact with the visuospatial executive task. Panel **(B)** presents a snapshot of the recorded treadmill pressure mat and the trace of center of foot pressure displacement for a complete gait cycle. Panel **(C)** presents AP and ML COP time-series data for 3 steps. Maxima and minima of COP excursion for right and left steps are quantified, and use to compute swing and step times and step length.

Figure 1A presents the components of the treadmill platform. Participants stood on a treadmill at a viewing distance of 100 cm from an 80 cm computer monitor. The following tasks were performed while walking on a treadmill at 0.9 m/s: for 1 min:

- Walk only (single task condition),
- Walk while performing a Visuomotor (VM) task,
- Walk while performing a Visuospatial Executive Game (VEG) task.

As presented in Figure 1 the treadmill is instrumented with a pressure mat (Vista Medical, Canada) which was used to record vertical foot contact forces and to compute spatiotemporal gait variables (Betker et al., 2008). At a walking speed of 0.9 m/s, and duration of 1 min, then data for 30 consecutive steps were obtained. Before starting the tests participants walked for 5 min to acclimate to treadmill walking. Test 2 was conducted 1 week after test 1.

### Visuospatial executive tasks

A custom computer application with the following two assessment modules was used for this study: (a) a Visuomotor (head tracking) module and (b) a Visuospatial Executive game (VEG) module (Szturm et al., 2013a, 2015a). An inexpensive, commercial motion sense mouse (Gyrations, SMK-Link, USA) was used to control and interact with the visuospatial executive games. The motion-sense mouse is small with inertial sensors which are used to derive instantaneous angular position. The motion sense mouse allows head angular rotation to be translated and interpreted as a standard USB computer mouse. Velcro secures the wireless motion mouse to a headband, and with this simple method, the head rotation is used as the pointing device to control the position and motion of the computer game sprite. Therefore, a hands-free computer/game controller to interact with the game activities of the assessment software is introduced. Head pointing movements are among the most natural and can easily be performed with minimal instruction and by most people.

### Visuomotor (VM) task

The goal is to align two moving objects. One object, a bright circular object, is computer controlled and moved horizontally, left and right (cyclic motion) on a computer display for 45 s. Motion frequency was 0.5 Hz and amplitude was 70% of monitor width. The second object, a square, was slaved to head rotation using the head-mounted motion sense mouse (Szturm et al., 2015a). The goal of the task is to maintain an overlap of the two objects for 45 s. The computer application generates a logged data file to record the coordinates of the circle (target) and square (head rotation) at 100 Hz. The data file is processed off-line to quantify visuomotor performance as described below. Participants were tested in one direction, (a) horizontal motion (left/right).

### Visuospatial executive game (VEG) task

**Figure 2:**
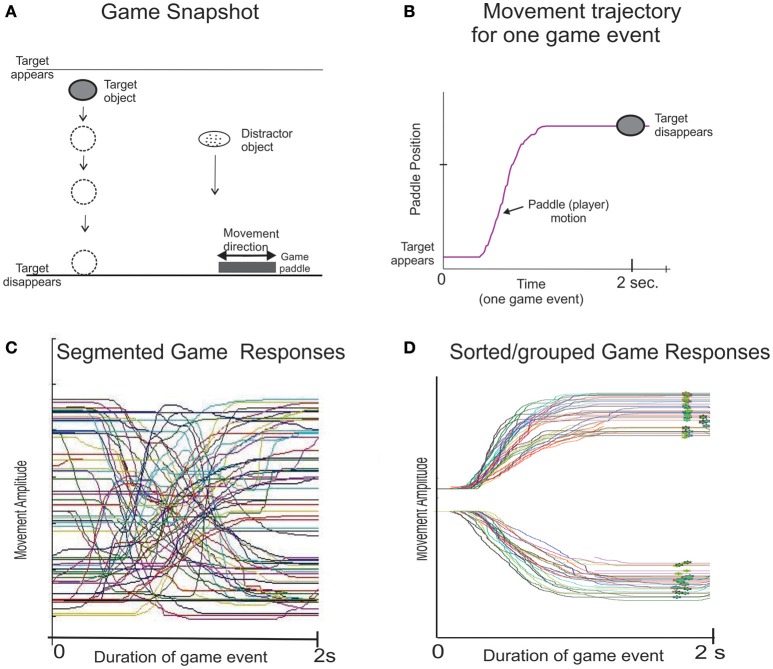
**Illustration of the game objects and motions of the visuospatial executive game**. Panel **(A)** Screenshot of the VEG game software. The target appears at the top of the screen at a random location and the participant interacts with the target. Panel **(B)** presents the trajectory for one game movement response from target appearance to target disappearance panel **(C)** presents overlay trajectories of all game movement responses (as shown in panel **B**) for one game session of 60 s duration. Some game movements are upward and some downward (i.e., different movement directions), and some game movements are medium and some large amplitudes. Panel **(D)**, the game movement responses of panel **(C)** are sorted and grouped in bins, which in this case represent medium amplitude movement responses for both directions; leftward game movements (upward trajectories), and rightward game movements (downward trajectories).

The goal was to move a paddle (the game sprite) to interact with moving game objects. Head rotation via the motion sense mouse was used to move the game paddle and catch the target objects while avoiding distractor objects. See Figure 2A for illustration of the game function. The target object was a brightly colored circle and the distractor object was an oval shaped object. The target and distractor objects appear at random locations at the top of the monitor and moves to the bottom in a time period of 1.5 s and then disappear. For each game event (target appearance) the participant moves a game paddle along the bottom of the display to catch the target object and avoid any distractor objects. The game was played for 60 s or 45 game events. The software indexes the “times” for the appearance and disappearance of each target game object and logs the position coordinates of the game objects and game paddle (participant's head rotation) at a sampling rate of 100 Hz. The data file is processed off-line to quantify visuospatial executive performance as described below.

The visuomotor and visuospatial executive game tasks were performed in standing (baseline and during treadmill walking (dual-task condition). Prior to testing, the participants were allowed to play the tracking and game tasks while sitting for a few minutes to become familiar with each task.

The treadmill walking tasks were difficult when performing the concurrent game tasks. The treadmill was equipped with safety side rails in easy reach, and participants were fitted with a safety harness secured above to a support system. Also during all test a Physical Therapist stood behind the participants to provide assistance if required.

## Data analysis

### Spatiotemporal gait variables

The average and coefficient of variation (COV) over 30 consecutive steps were determined for (a) right step time, (b) right single support times, and (c) right step length. Step time is defined as the time from a right foot off (beginning of left single support) to left foot off. Note analysis was also performed for left steps. Since the statistical analysis showed no significant difference in means of right and left gait variables, only the right gait variables will be presented.

### Visuomotor performance

**Figure 3:**
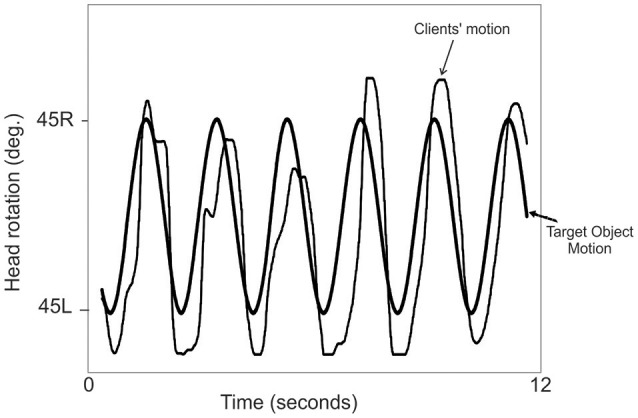
**Presents Synchronous plots of the target cursor motion and user movement trajectories (head rotation) for a typical VM tracking task**. Maxima are the left most position and minima the right most position.

Figure 3 presents synchronous plots of the target motion (circle) and user head rotation (square) for a typical visuomotor task. A sine-wave function of the reference target cursor waveform was determined, Head rotation trajectories were fit to the sine-wave function, and the coefficient of determination (COD) was computed based on total and the average residual difference between the position (pixel coordinates) of the target and head cursor for all sampled data points. The first two cycles of the tracking tasks were excluded to allow the participants' time to acquire the moving target and begin tracking. MATLAB (The Math Works, Natick, MA, version 2010a) was used to compute the COD.

### Visuospatial executive performance measures

Figure 2B presents the trajectory of an individual game movement response. Each game event was 1.5 s in duration from target appearance to target disappearance. Figure 2C Presents overlay trajectories of individual game movement responses in one game session. Based on time indices of target appearance and disappearance the software segments all game movement traces for each direction and game movement amplitude. The software then sorts these movement traces by direction and for medium amplitude movements (Figure 2D). Thus, the software produces multiple, standardized contextual movement events (Players actions) for each direction. For a detailed description of the game movement indexing and segmentation see (Lockery et al., 2011; Szturm et al., 2015a). The following variables were quantified; (a) success rate determined as the percentage of target objects that were caught, (b) average Response Time: the time from target appearance to the start of the game paddle movement and (c) average Movement Time, the time from start of the game paddle movement to the time it reaches its plateau at the point the target disappears Response Time and Movement Time were averaged over all game movement responses separately for leftward and rightward directions. Statistical analysis (paired *t*-test) revealed no significant difference in average response time or average movement time between leftward and right game movement responses, and therefore only averages for leftward game movements are presented in the present study.

### Statistical analysis

Relative reliability was assessed using a fixed model intra-class correlation coefficient (ICC) (Weir, 2005). The ICC scores were interpreted as high when equal to or greater than 0.70, as moderate between 0.5 and 0.69, and as low when less than 0.50 (Lexell and Downham, 2005). Absolute reliability was analyzed using standard error of measurement (SEM). Systematic errors between the test periods were evaluated using a paired *t*-test. Normality of data was assessed using the Shapiro-Wilks test (*n* < 50). This test revealed a normal distribution for all variable (*p* > 0.1).

Discriminative validity was evaluated using a paired *t*-test to examine the effect of dual-task conditions on; (a) gait performance and (b) visuomotor and visuospatial executive performance. For the VM and VEG performance measures, the single task condition is when performed in standing on a fixed surface and the DT condition is when performed during treadmill walking.

SPSS software for Windows, version 20.0 (SPSS Inc. Chicago) was used for all statistical analysis procedures.

## Results

Nineteen females and six males participated. The median age was 65, range 61–68 years. Group average gait speed was 1.1 m/s and a standard deviation (SD) of 0.14, and the average distance walked in 6 min was 532 m and SD of 87. All participants walked outdoors on a regular basis, and none had reported a fall in the last year.

Table 1 presents the results of the test-retest reliability analyses for the temporal and spatial gait variables. With a few exceptions, high ICC values of 0.71–0.85 were observed for both averages and COV. Moderate ICC values were observed for average step length (0.65) during the visuomotor task, and for SL COV (0.65) when performing the executive game task. The standard error of measurement (SEM) as a percentage of the group mean values ranged from 4 to 15% and was less than 10% in the majority of cases. Based on a paired *t*-test analyses, no systematic errors in the average or COV variables were observed between the two test sessions for either the single or the two DT walk conditions. As presented in Table 2, moderate ICC values of 0.6–0.65 were observed for visuomotor and executive game performance measures. The SEM as a percentage of the group mean values for the visuomotor task was 15%, and for the executive game performance measures, it ranged from 5 to 11%. The results of the paired *t*-tests revealed no significant difference in visuomotor or executive performance measures between the two test periods.

**Table 1:** **Results of statistical analysis, ICC scores, standard error of measurement (SEM), group means and 95% confidence interval (CI) for the spatial and temporal gait parameters during the three walking conditions**.

**Task conditions**	**Test 1 Mean (95%CI)**	**Test 2 Mean (95%CI)**	**ICC**	**SEM**
**WALK ALONE**				
Avg-SsT, ms	376 (359.6–392.4)	386 (369.3–402.7)	0.8	19.1
Avg-SwT, ms	457 (429.6–484.4)	478 (450.6–505.4)	0.7	40.7
Avg-SL, cm	40.4 (38.1–42.7)	41.6 (39.7–43.5)	0.8	2.9
COV-SsT	10.4 (9.48–11.3)	10.7 (9.9–11.5)	0.8	1.1
COV-SwT	12.1 (10.6–13.6)	11.5 (10–13)	0.9	0.5
COV-SL	14.7 (13.1–16.2)	15.3 (13.9–16.8)	0.8	1.6
**DT-VM**				
Avg-SsT, ms	401 (379.6–422.4)	393 (376.2–409.4)	0.8	30.3
Avg-SwT, ms	437 (409.6–464.4)	421 (393.6–448.4)	0.8	36.1
Avg-SL, cm	32.5 (30.7–34.3)	31.8 (30.3–33.3)	0.7	2.6
COV-SsT	13.2 (12–14.4)	13.7 (12.2–15.2)	0.7	1.5
COV-SwT	12.7 (11.5–13.9)	11.5 (10.3–12.7)	0.7	1.7
COV-SL	16.2 (14.7–17.7)	16.3 (15.2–17.4)	0.9	1.2
**DT-VEG**				
Avg-SsT, ms	445 (422.2–467.8)	436 (419.7–452.4)	0.7	35.1
Avg-SwT, ms	294 (281.2–306.8)	302 (288.1–315.9)	0.7	18.3
Avg-SL, cm	32.5 (30.7–34.3)	32.5 (30.8–34.2)	0.8	4.1
COV-SsT	16.2 (14.3–18.1)	19.4(17.3–21.5)	0.8	2.1
COV-SwT	19.8 (18.9–20.7)	20.0 (17.7–22.3)	0.7	1.3
COV-SL	23.9 (22.3–25.5)	23.1 (21–25.2)	0.8	2.1

*SsT, single support time; SwT, swing time; SL, step length; Avg, Average; COV, coefficient of variation (%)*.

**Table 2:** **Results of statistical analysis, ICC scores, standard error of measurement (SEM), group means and 95% confidence interval (CI) for visuomotor and Visuospatial Executive Game tasks during treadmill walking**.

**Outcome measures**	**Test 1 Mean (95%CI)**	**Test 2 Mean (95%CI)**	**ICC**	**SEM**
**VISUOMOTOR**				
COD	0.6 (0.6–0.7)	0.7 (0.6–0.7	0.7	0.1
**VISUOSPATIAL EXECUTIVE GAME**				
Success Rate, %	82.5 (79.5–85.5)	84 (83.3–88.7)	0.7	3.6
Avg-Response Time, ms	502 (475.5–528.5)	493 (465.9–508.1)	0.6	41
Avg-Movement Time, ms	526 (505.7–552.3)	514 (482.5–535.5)	0.6	56

**Figure 4:**
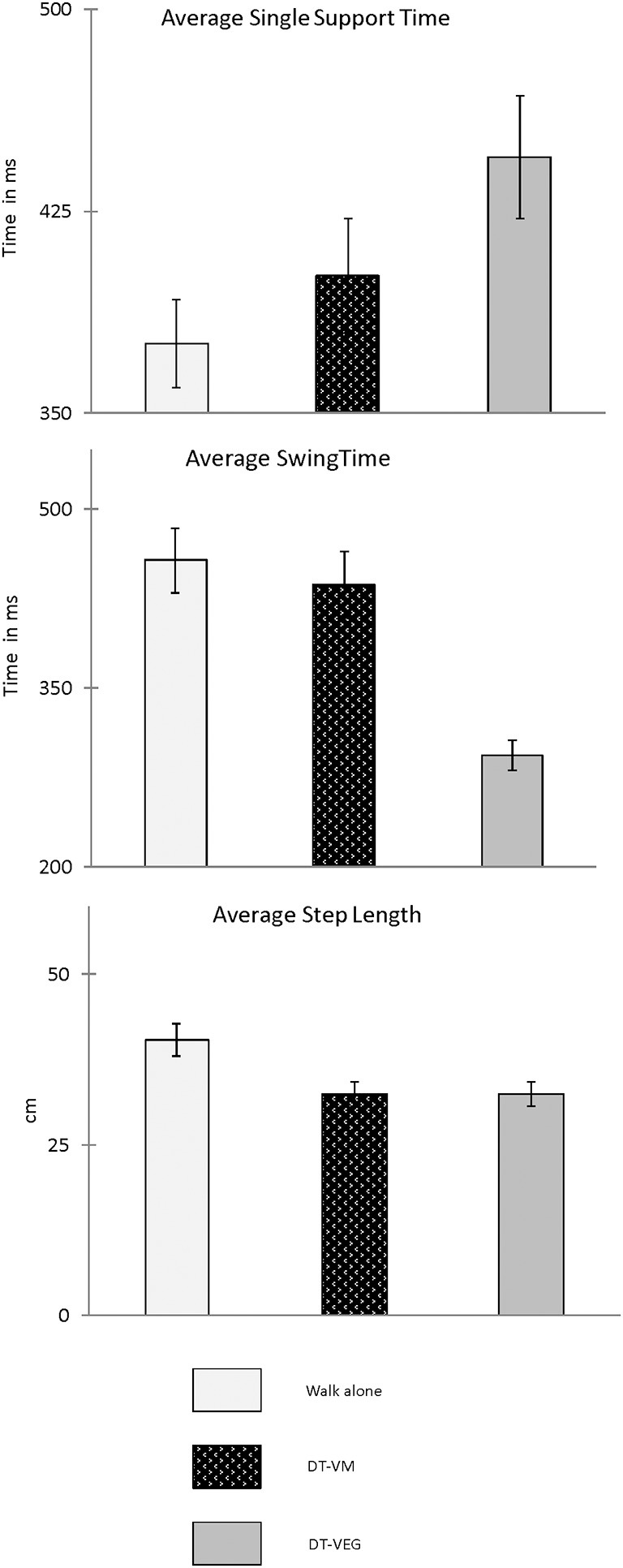
**Presented are group means and 95% confidence intervals (CI) for the average spatiotemporal gait variables (SsT, SwT, SL) obtained during walk only and dual-task walk conditions**.

**Figure 5:**
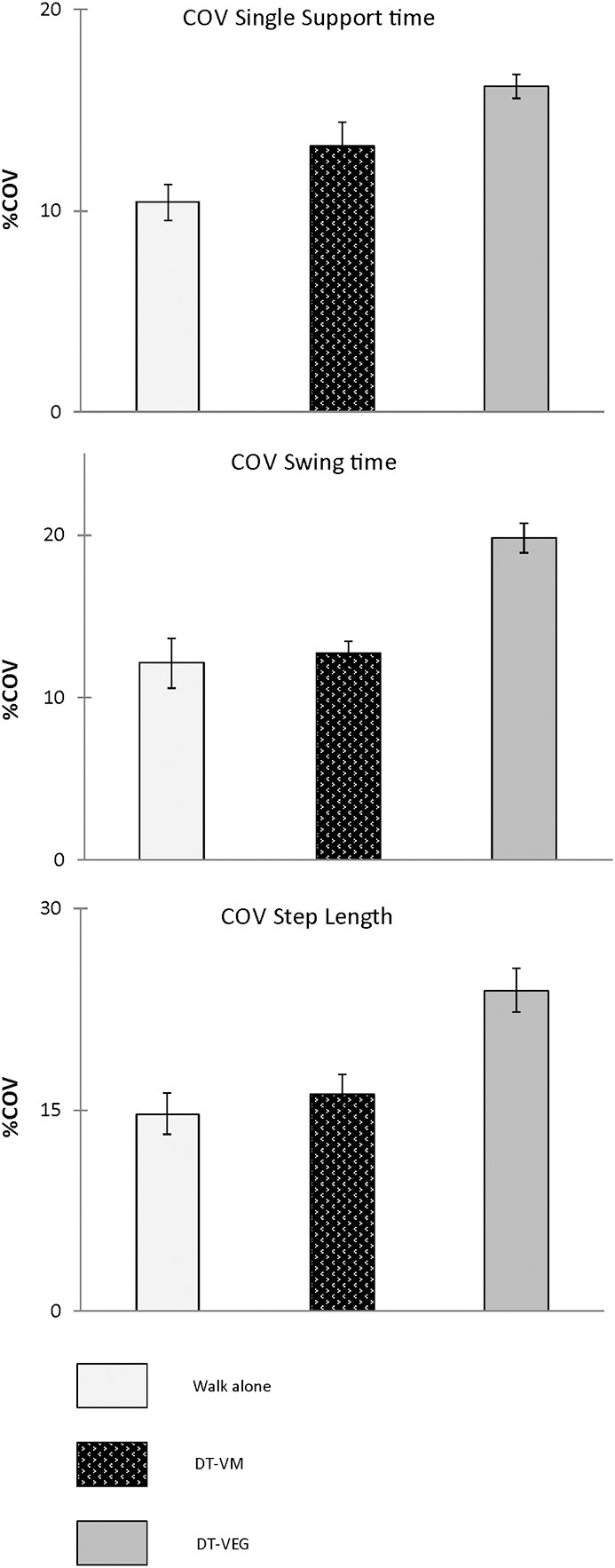
**Presented are group means and 95% CI for COV of the spatiotemporal gait variables (SsT, SwT, Sl) obtained during walk only and dual-task walk conditions**.

Group means and 95% CI for average and COV of spatiotemporal gait variables obtained during walk only and dual-task walk conditions are presented in Figures 4, 5 respectively. As presented in Table 3 all temporal and spatial gait variables did demonstrate a significant change when performing the visuospatial executive tasks (DT condition) as compared to walk alone (single task condition). Average swing time (*p* < 0.01) and average step length (*p* < 0.001) significantly decreased from single to dual-task conditions, whereas average single support time significantly increased (*p* < 0.001) from single to dual-task conditions. Group means and 95% confidence intervals (CI) are presented in Table 1. There was a significant increase in COV for all gait variables when performing the visuospatial executive game task as compared to walk alone (*p* < 0.01). As presented in Table 3 the majority of gait variables did not demonstrate a significant change when performing the visuomotor task. There were two exceptions; a significant decrease in Average step length (*p* < 0.001) and a significant increase in single support time COV (*p* < 0.001) as compared to walk alone.

**Table 3:** **Effect of dual-tasking on spatiotemporal gait variables (Average and COV)**.

**Gait variables**	**Walk alone vs. Walk + visuospatial task**	**Walk alone vs. Walk + visuomotor task**
	***t*****-statistics, *p*-value**	***t*****-statistics, *p*-value**
Avg-SsT	4.5, 0.01	1.9, 0.07
Avg-SwT	10.1, 0.01	1.0, 0.3
Avg-SL	8.5, 0.01	6.8, 0.01
COV-SsT	5.4, 0.01	3.7, 0.01
COV-SwT	8.1, 0.01	0.6, 0.5
COV-SL	7.8, 0.01	1.4, 0.18

*Degrees of Freedom (df) = 24*.

**Figure 6:**
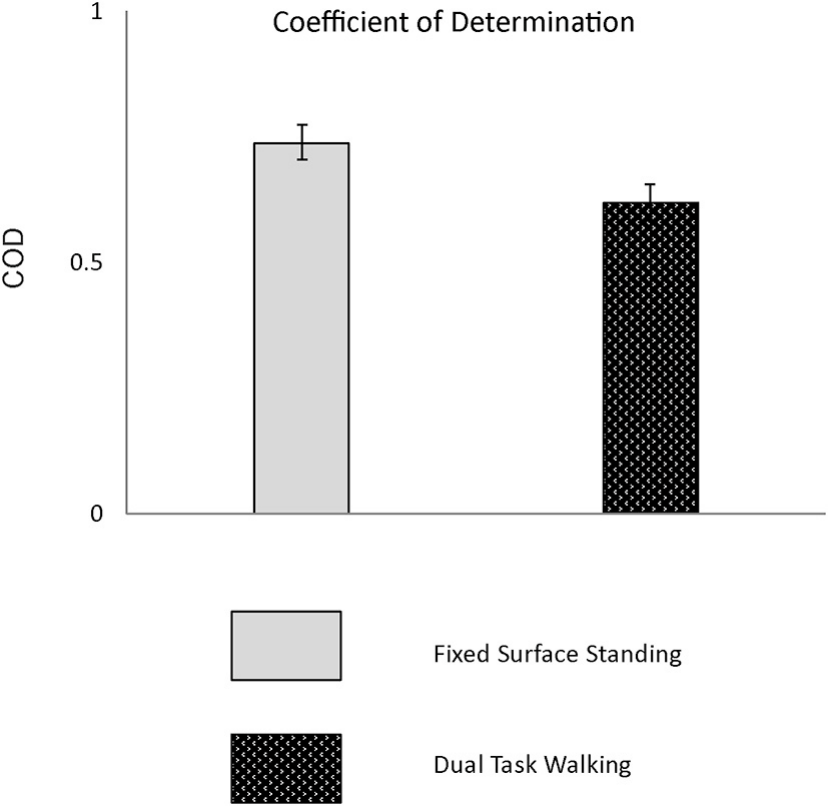
**Presented are group means and 95% CI for the visuomotor performance measure obtained during fixed surface standing and dual-task walk conditions**.

Figure 6 presents the group means and 95% CI for the visuomotor performance measure obtained during walk only and dual-task walk conditions. As presented in Table 4 there was a significant decrease in visuomotor performance when tested during treadmill walking as compared to standing; (*p* < 0.001). Group means and 95% confidence intervals (CI) are presented in Table 2. When tested in standing visuomotor performance was 0.78 and decreased to 0.65 during walking.

**Table 4:** **Effect of dual-tasking on Visuomotor and Visuospatial executive game performance measures**.

**Outcome measures**	***t*****-statistics, *p*-value**
**VISUOMOTOR TASK**	
Coefficient of Determination (COD)	4.5, 0.01
**VISUOSPATIAL EXECUTIVE TASK**	
Success Rate, %	2.3, 0.04
Avg-Response Time, ms	0.1, 0.9
Avg-Movement Time, ms	0.9, 0.4

*Degrees of Freedom (df) = 24*.

**Figure 7:**
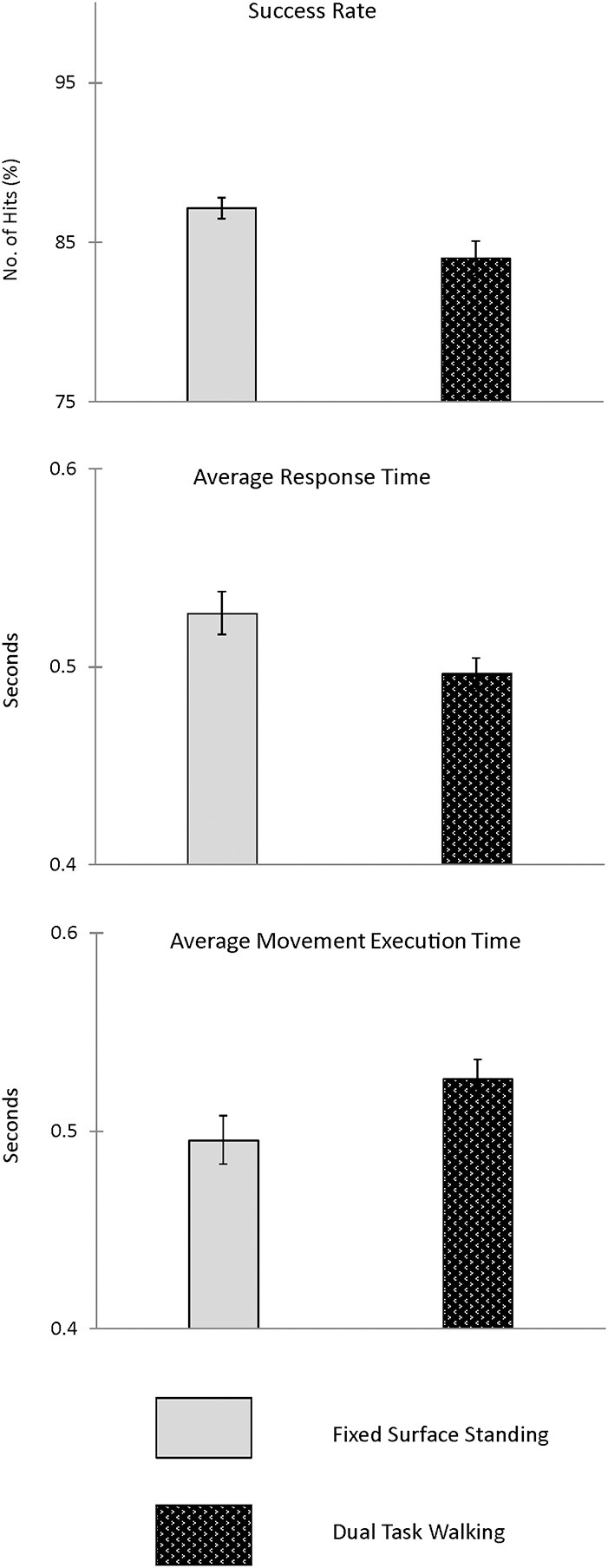
**Presented are group means and 95% CI for the visuospatial executive performance measures obtained during fixed surface standing and dual-task walk conditions**.

Figure 7 presents the group means and 95% CI for the visuospatial cognitive performance measures obtained during walk only and dual-task walk conditions. As presented in Table 4 there was a significant decrease in visuospatial executive performance when tested during treadmill walking as compared to standing. There was a significant decrease in success rate (*p* < 0.04), but no significant change in Response time or Movement Time when tested during treadmill walking as compared to standing. Success rate, when tested in standing, was 94%, as compared to 82% during walking.

## Discussion

High ICC values (greater than 0.8) have been reported for average gait variables during overground and treadmill walking (Brach et al., 2008; Paterson et al., 2008). In the present study comparable high to moderate ICC values were observed for all average gait variables during walk alone trials. The present results extend reliability analysis to include DT treadmill walking involving computerized visuomotor and visuospatial executive tasks. A number of studies have reported low ICC values (less than 0.4) for measures of gait variation (Brach et al., 2008; Paterson et al., 2008; Faude et al., 2012). Immediate test re-test reliability was examined in older adults (mean age of 75.5 years) during an over ground DT walking test consisting of counting backward (Beauchet et al., 2011). Low ICC values of 0.28 for stride variability were observed. In contrast, the present results show modest to high ICC values for COV of gait variables during DT treadmill walking. There are a few important differences between the present study and the studies using over ground walking. In the present study participants viewed a computer monitor and performed standardized visual-spatial executive tasks for a duration of 60 s. The VEG tasks employed in the present study were executively demanding, requiring timely responses (less than 1 s) to identify a moving object as the target or a distractor, to estimate its final position, and to move the game sprite using head rotation in order to intercept the moving target, i.e., accuracy requirement. Secondly, walking speed was controlled and over 30 consecutive steps were recorded, as opposed to recordings of a small number of consecutive steps on a short walkway (i.e., 3–5 m). Many studies have demonstrated that spatiotemporal gait variables are influenced by walking speed (Kang and Dingwell, 2008; Stoquart et al., 2008; Keene et al., 2016). Furthermore, it has been shown that using a continuous walking protocol instead of short intermittent walks, and collecting more than 30 steps improved reliability, in particular for measures of gait variability (Galna et al., 2013). Low between day ICC values (less than 0.4) for gait variation has been reported for active, healthy older adults (mean age of 64 years) when tested on a treadmill (Faude et al., 2012). It is not clear why this large difference (during walk alone condition) is observed between the present study and the results of (Faude et al., 2012). One difference is the walking speed of the two studies; 1.3 vs. 0.9 m/s. A lower speed was used in the present study because the addition of the visuospatial executive activities did make the walking more difficult.

The present results show a small absolute variability; SEM ranged from 4 to 15% of the mean scores and the majority were less than 10%. Taken together the moderate to high ICC values and the small measurement error indicate that this tool has the ability to repeatedly record reliable data from active older adults, and would serve as acceptable outcome measures to examine effects of preventative measures and targeted interventions on dual-task walking performance.

Ability to recover from a sudden loss of balance is only part of the equation that governs the probability of falling; also important is what causes the instability. Besides large physical disturbances, performing a concurrent executive task can lead to a sudden change in locomotor rhythm and even falls. A number of studies have examined the interaction among physical and information processing load as a function of aging using a DT paradigm (Plummer-D'Amato et al., 2012; Xiangde, 2014). In the present study where speed is held constant all gait variables tested (average and COV) except one case were significantly affected when performing the visuospatial executive task as compared to walk alone. Average Single support time did increase when the information processing load was added. These findings for average gait variables would indicate the main effect of visuospatial processing load on locomotor rhythm. There was also a significant increase in COV for all gait variables when the visuospatial load was increased. These results are consistent with other studies which have examined dual-task effects on gait variation during overground walking in healthy young and older adults (Ijmker and Lamoth, 2012; Montero-Odasso et al., 2012). Variation of gait variables has often been used to index gait stability (Baltadjieva et al., 2006; Herman et al., 2010; Lord et al., 2011). Herman et al. (2010) reported swing time variability to be nearly 20% higher in fallers compared to non-fallers. Unlike the dual-task visuospatial trials, there was little change in gait performance during the dual-task visuomotor trials. The VM task has very simple executive demand, overlap two visible objects. This may explain why the dual-task VM trials had little effect on spatiotemporal gait variables.

A significant decrease in the Success Rate of the visuospatial tasks was observed during treadmill walking. However, Response Time and Movement Time were not affected by the increased processing demands of treadmill walking. Motor planning and temporal parameters of precision movements would not be the only factors that contribute to movement accuracy of the visuospatial task. The estimation of the final target position and control requirements of the head rotations would also contribute to movement accuracy. This is an area that will receive further investigation. The performance of the visuomotor task also decreased during treadmill walking as compared to standing. The VM task required continuous visual attention and foveation to determine the relative positions (overlap error) of 2 moving objects, and this spatial feedback would be required to maintain or restore their overlap. The significant decline in VM performance is likely due to the increase in the amount of passive head movement between standing vs. treadmill walking. There is a considerable increase in the magnitude of passive head velocity during walking, as much as 10 times of that seen during standing (Szturm et al., 2015b). Passive head motion will cause increased retinal image slip, and thus affects the ability to stabilize gaze during continuous fixation tasks, such as, tracking and interacting with moving targets (Scherer et al., 2008; Lambert et al., 2010).

Most commonly dual-task studies have utilized executive tasks, like walking while talking, verbal fluency or number subtraction that is typically only assessed qualitatively, do not involve visuospatial processing, and are limited in what individual brain areas are recruited (Al-Yahya et al., 2011). Visual attention, tracking, choice responses and the processing of object locations/trajectories and their spatial relations with respect to other objects are key aspects to consider in locomotor control and are important factors in fall risk (Bagurdes et al., 2008; Nagamatsu et al., 2009; Murray et al., 2010). The present computerized dual-task protocols broaden the types of standardized executive activities for use with treadmill walking that has previously been reported.

### Study limitations

Treadmill walking does constrain gait, for example, by the belt width, and does not reflect all aspects of over ground walking behavior (Hollman et al., 2016). The present visuospatial computer task involves both head rotation and information processing, and at this point we cannot rule out any intersegmental mechanical effect of the head rotation as a cause of the gait changes observed between the walk alone and the DT walk trials. Head rotations during the VEG task were relatively small and slow i.e., the majority of the head rotations for game responses were less than 20° and movement duration was in the order of 500 ms. Therefore, every 2 s the participant produced these small ramp head rotations. The head movements were rotations so the mass center of the head segment would not change relative to body center of mass. Duysens et al. examined COP migration during an open-loop tracking tasks (up to 30° of visual target motion) while treadmill walking (Duysens et al., 2008). Three tasks were performed; tracking with eye movements only (head stationary), tracking by rotating the head in synchrony with the moving visual target (open-loop tracking task), and tracking while rotating the trunk in synchrony with the moving visual target. The results demonstrated no significant deviation of the COP migration when participants performed the tracking task with eye or head rotation, whereas, trunk rotations led to a doubling of ML-COP deviation. The mechanical effect of head rotation on gait rhythm pacing and variation will receive further investigation.

## Conclusion

This study demonstrates the reliability and reproducibility of the computer-based assessment tool for DT treadmill walking. The high to Moderate ICC values, the small standard errors of measurement and the lack of systematic errors in the measures indicate that this tool has the ability to repeatedly record reliable data from community-dwelling older adults Improved and affordable methods of screening and fall risk assessment in the community particularly are important because continued difficulties and fall injuries will have a sizable impact in this population.

## Ethics statement

Bannatyne Campus Health Research ethics board at the University of Manitoba (Ethics Reference number H2011:284) approved the study “Computerized Dual-task testing of gait and visuospatial cognitive functions; Test-retest reliability and validity.” A written informed consent was acquired from all participants before the screening process by blind assessors. This study was carried out in accordance with the recommendations of “University of Manitoba, Bannatyne campus Health Research Ethics Board” with written informed consent from all subjects. All subjects gave written informed consent in accordance with the Declaration of Helsinki. The protocol was approved by the “University of Manitoba, Bannatyne campus Health Research Ethics Board.”

## Author contributions

TS and VS: study concept and design; acquisition, analysis, and interpretation of data; preparation of the manuscript. AK: Acquisition, analysis, and interpretation of data; preparation of the manuscript. MN: Analysis, and interpretation of data; preparation of the manuscript.

### Conflict of interest statement

The authors declare that the research was conducted in the absence of any commercial or financial relationships that could be construed as a potential conflict of interest.
